# Do CS-US Pairings Actually Matter? A Within-Subject Comparison of Instructed Fear Conditioning with and without Actual CS-US Pairings

**DOI:** 10.1371/journal.pone.0084888

**Published:** 2014-01-23

**Authors:** An K. Raes, Jan De Houwer, Maarten De Schryver, Marcel Brass, Raffael Kalisch

**Affiliations:** 1 Ghent University, Ghent, Belgium; 2 Erasmus University Rotterdam, Rotterdam, The Netherlands; 3 Institute for Systems Neuroscience, University Medical Center Hamburg-Eppendorf (UKE), Hamburg, Germany; 4 Neuroimaging Center Mainz (NIC), Focus Program Translational Neuroscience (FTN), Johannes Gutenberg University Medical Center, Mainz, Germany; Universidad de Granada, Spain

## Abstract

Previous research showed that instructions about CS-US pairings can lead to fear of the CS even when the pairings are never presented. In the present study, we examined whether the experience of CS-US pairings adds to the effect of instructions by comparing instructed conditioning with and without actual CS-US pairings in a within-subject design. Thirty-two participants saw three fractals as CSs (CS^+^1, CS^+^2, CS^−^) and received electric shocks as USs. Before the start of a so-called training phase, participants were instructed that both CS^+^1 and CS^+^2 would be followed by the US, but only CS^+^1 was actually paired with the US. The absence of the US after CS^+^2 was explained in such a way that participants would not doubt the instructions about the CS^+^2-US relation. After the training phase, a test phase was carried out. In this phase, participants expected the US after both CS^+^s but none of the CS^+^s was actually paired with the US. During test, self-reported fear was initially higher for CS^+^1 than for CS^+^2, which indicates that the experience of actual CS-US pairings adds to instructions about these pairings. On the other hand, the CS^+^s elicited similar skin conductance responses and US expectancies. Theoretical and clinical implications are discussed.

## Introduction

The publication of Rachman's three-pathway theory on the etiology of anxiety disorders [1] greatly stimulated research on the acquisition of fear. In this theory, Rachman identifies observation and instruction as important sources of fear next to the direct experience of pairings between originally neutral stimuli (conditioned stimuli; CSs) and aversive stimuli (unconditioned stimuli; USs). For instance, someone might develop fear for spiders after observing another person's panic in response to spiders (learning by observation) or after being told by that the bites of certain spiders are lethal (learning by instructions). These ideas have been supported by several retrospective studies in which observation and instruction were identified as common sources of fear in children [2]–[5].

Experimental studies further confirmed the role of observation and instruction in the etiology of fear (see [6], and [7], for reviews on these topics). For instance, Field and colleagues told children that “Once upon a time, there lived a horrible scary monster called Makis. Makis was 12ft tall with huge sharp fangs for eating children with” [8] (p.1273). They observed that these instructions led to increases in reported fear of the previously unknown stimulus [8], negative implicit associations with that stimulus [9], [10], increased physiological responding to that stimulus [11], and avoidance [9], [11].

The studies of Field demonstrate that fear for a previously unknown stimulus can be acquired based on instructions about the properties of the stimulus (i.e., threatening information about the stimulus). In addition, experimental fear conditioning studies have shown that fear conditioning can result not only from the experience of CS-US pairings but also from mere instructions about the presence of those pairings in the future. For instance, Olsson and Phelps (p. 825) showed participants a neutral face and told them that they would “receive at least one and at most three shocks paired with this face” [12]. This instruction led to enhanced physiological responding to that face (see also the seminal work of [13]).

So far, only two studies have looked at the joined impact of direct experience and instructions [14], [15]. In the study of Field and Storksen-Coulson [14], 6- to 8- year-old children received threatening information about an unknown animal. After this, they had a negative experience with either this animal or a control animal. The effect of a negative encounter with a novel animal was stronger when it was preceded by threatening information about this animal than when no information had been provided, while the effects of only a negative encounter or only threatening information were similar. These results are consistent with conditioning theories of phobias [16]–[18] which state a traumatic experience yields stronger effects when the experience concurs with previous beliefs on the CS-US contingency (i.e., compare the effect of a car crash in someone who is already convinced that cars are dangerous or in someone who thinks cars are generally safe). On the other hand, a similar study in adults, in which participants received computerized aversive CS-US pairings preceded either by threat information or no threat information on the CS, did not yield additive effects for the combination of experience and threat information [15]. That is, participants who had received threatening information about the CS showed higher initial fear beliefs about the CS at the start of conditioning, but actual CS-US contingencies did not further enhance these beliefs.

It still remains to be determined, however, whether actual CS-US encounters actually add anything to instructions about the CS-US contingency. To answer this question, one needs to manipulate the presence of actual CS-US encounters while controlling for the effect of instructions, CS and US experience and the predictive power of the CS (CS-US contingencies). No prior study meets these requirements. For instance, in the studies described above [12], [14], [15], all participants received actual CS-US pairings for each of the CSs. Moreover, in those studies, participants were given general threat information about the CS (e.g., “this stimulus is dangerous”) [14], [15] rather than instructions about a specific CS-US contingency (e.g., “this stimulus will be followed by a shock”).

Intuitively, most of us would probably be inclined to believe that experience of a CS-US pairing does add to the effect of mere instructions about this pairing. Still, from a theoretical viewpoint, this is not an obvious question to answer, nor is it a question with an evident answer. Most importantly, existing models of associative learning allow for the possibility that instructions about pairings lead to changes in behavior but are unconstrained as to whether experience would add anything to the effect of instructions.

First, association formation models posit that conditioned responding depends on the formation of links in memory (e.g., “A–B”; see [19]–[21]). Typically, these links are assumed to stem primarily from actual trial-by-trial experience e.g. [20], [21]. Nevertheless, some proponents of these models have argued that links in memory can be established in the absence of an actual US and even an actual CS (e.g., Field, 2006, p.864) [22]. Their main point is that evoking a representation of the CS-US contingency, for instance through instructions, can be sufficient to install links in memory and thus conditioned responding. However, it remains unclear whether a subsequent encounter with the actual CS-US contingency would further strengthen the CS-US link or whether instructions alone can suffice to install CS-US link of asymptotic strength.

Second, propositional models postulate that associative learning is mediated by the formation of propositions (i.e., qualified statements about relations between events; e.g., “A predicts B” or “A causes B”; see [23], [24]). These models assert that propositions can be formed both on the basis of direct experience of the CS-US pairs *and* on the basis of instructions about the CS-US relation. Therefore, they would have little difficulty to accommodate to the finding that experience does not add to instructions, provided that instructions lead to a clear proposition with high truth value [23]. Still, propositional models can also be instantiated in such a way that they would predict an added effect of actual CS-US pairings. For instance, they could argue that direct experience of a CS-US contingency enhances the truth value of a proposition relative to purely instructed contingencies and therefore strengthens the conditioned changes in behavior [23].

The fact that both propositional and association formation theories can handle both types of results indicates that neither theory is precise enough to derive specific predictions in this context (see also [25]). Therefore, examining whether actual CS-US pairings add to instructed conditioning would help to further constrain associative learning theories and force them to make explicit and testable assumptions about how the effect of instructions and experience interact.

Examining the added value of actual CS-US pairings can advance not only our theoretical understanding of (fear) conditioning, but can also increase our insight in the development and treatment of anxiety disorders. Previous research confirmed that there are different pathways to the development of anxiety disorders [5], [26]. If, however, we observe that the actual experience of CS-US pairings has effects on fear over and above the effects of instructions, it would suggest that the different pathways to fear are not entirely equivalent. Such a conclusion would strengthen the idea that the treatment of anxiety disorders should take into account the pathway via which fear originated. On the other hand, if we find a strong effect of instructions without evidence for the added value of experiencing CS-US pairings, this would speak to the importance of the verbal pathway and thus of prevention measures that are directed at this pathway, such as alerting the parents of vulnerable children to the adverse consequences of providing threatening information with stimuli such as small animals, water, or heights [7].

In our study, we used a within-subjects design that allowed us to manipulate the presence of the actual CS-US pairing while controlling for CS experience, US experience, and instructions. At the start of the experiment, participants received the same CS-US instructions for two CS^+^s (CS^+^1 and CS^+^2). They were told that both CS^+^s would be followed by a shock (US), whereas the CS^−^ would never be followed by shock. However, only CS^+^1 was actually paired with the US. To ensure that participants believed CS-US instructions for both CS^+^1 and CS^+^2, we included a training phase during which CS^+^1 was paired with the US whereas CS^+^2 was paired with a placeholder (picture of a lightning bolt) instead of the US. The number of CS-US and CS-placeholder pairings was the same. Through written instructions, we explained that the goal of this phase was to get participants acquainted with the experiment procedure. We told participants that we did not want to expose them to too many shocks during training and therefore, one stimulus (CS^+^2) would be followed by a placeholder instead of the actual US. By giving participants an explanation for the absence of the US after the CS^+^2, we hoped that they would not simply dismiss the instructions about the CS^+^2-US relation as invalid but would continue to evaluate the instructions for CS^+^1 and CS^+^2 as equally valid. Because CS^+^1 and CS^+^2 differed only in terms of actual pairing with the US and not in terms of instructions, we will refer to the CS^+^2 as the *merely instructed* CS.

After the training phase had ended, participants were instructed that the experiment would now start for real and that, from now on, all shocks (USs) would be actually delivered. During this phase, from here on referred to as the test phase, no USs or placeholders were actually delivered, however. This was important to assure that participants would never experience the CS^+^2 being paired with the US. The test phase was the critical phase for contrasting responses to a CS for which actual CS-US pairings had been delivered (CS^+^1) to responses to a merely instructed CS (CS^+^2).

During both training and test, responses to both CS^+^s were contrasted with responses to a CS^−^ (i.e., a stimulus that was never paired with the US or instructed to be followed by the US). At fixed time intervals during both the training and the test phases, participants were asked to report US expectancy and fear for both CS^+^s and the CS^−^. As such, we assessed participants' cognitive expectancy of the US (US expectancies) as well as their affective status (self-reported fear) in response to the CSs. In addition to these ratings, skin conductance responses (SCRs) were measured on a trial-by-trial basis

Because participants were clearly informed that during training, only CS^+^1 would be paired with the US, we expected that the CS^+^1 would elicit more pronounced SCRs, US expectancy and fear than the CS^+^2 and the CS^−^ during this phase. As mentioned above, the test phase was the crucial phase in which we tested whether actual CS-US pairings added to instructions on the CS-US contingencies by contrasting responses to CS^+^1 and CS^+^2. If experience of actual CS-US pairings matters, self-reported fear, US expectancy, and SRC in response to CS^+^1 should still be enhanced relative to CS^+^2 during the test phase.

## Methods

### Participants

Thirty-two undergraduate students registered for the study through the on-line system of participant recruitment (Experimetrix) of Ghent University. The sample was predominantly female (6 men). Mean age was 21.81 (*SD* = 2.18). Participants received eight Euros in exchange for participation. Ethical approval for this study was obtained by the Ethic Committee of the Faculty of Psychology and Educational Sciences of Ghent University.

### Material

#### Apparatus

Hardware consisted of two PCs and a Coulbourn Lablinc V (Coulbourn Instruments, Allentown, PA). One PC controlled the experiment through Inquisit 3.0 (Millisecond Software). The other PC was used to display and collect the physiological data. The Inquisit PC was connected to two CRT screens. One CRT screen was placed in the main lab space and allowed the experimenter to follow the progress of the experiment. The other screen, a 1024×768 pixels CRT screen, was placed in a separate test room. Besides the CRT screen, the test room also contained the electrodes for the measurement of skin conductance responses, and a constant current stimulator to deliver shocks. An intercom system allowed communication between the experimenter in the main lab space and the participant in the test room.

#### Experimental stimuli

**Figure 1:**
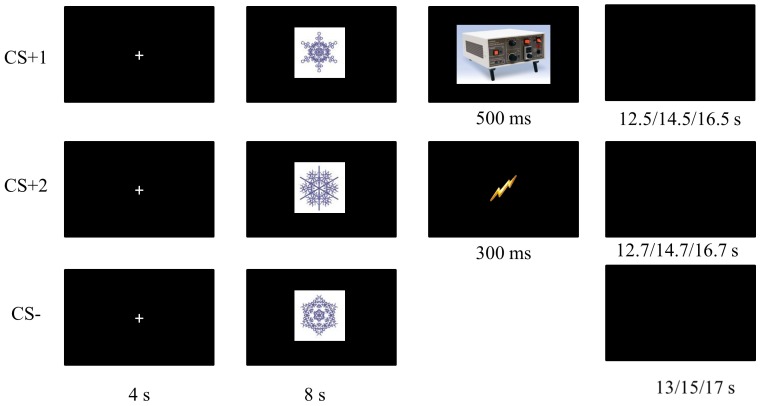
Overview of a reinforced CS^+^1 trial, a reinforced CS^+^2 trial and a CS− trial. Picture of stimulator signifies delivery of electric shock (US). Lightning bolt: delivery of US placeholder instead of US.

Three fractal figures (snowflakes) that were easily discernible served as conditioned stimuli. The allocation of these figures to the function of CS^+^1, CS^+^2 and CS^−^ was counterbalanced across subjects. The color of the fractals was blue. The fractals were placed on a white square surface of 200×200 pixels (see Figure 1).

The US was an electrical shock delivered by a constant current stimulator (DS7A, Digitimer, Hertfordshire, UK). This stimulus was administered by two lubricated Fukuda standard Ag/AgCl electrodes (1-cm diameter) to the right leg over the retromalleolar course of the sural nerve. Onset/offset and timing of the shock stimulus was controlled by a slave computer. The electrocutaneous stimuli consisted of a series of 38 rectangular pulses (2 ms in duration with an inter pulse interval of 6 ms) and had a total duration of 300 ms. The intensity of the stimulus was individually determined through a standard work-up procedure. The placeholder stimulus consisted of a 114×114 yellow drawing of a lightning bold that appeared in the center of the computer screen (see Figure 1).

#### Ratings

Self-reported CS fear and US expectancy were assessed for all CSs in separate ratings blocks interspersed between conditioning trials. These ratings were performed on screen. On a typical rating trial, the CS was presented centrally, while the question on fear or US expectancy was situated on top of the CS and a rating scale was presented below. Before each rating phase, participants were instructed to respond to the questions that would appear at the top of the screen through selecting the response possibility that felt most appropriate to them. Furthermore, it was stressed that these questions pertained to *their most recent encounter* with the CSs during the foregoing (conditioning) phase. In addition, participants were instructed that “if you are asked about your expectancy of the electrical shock, we refer to the actual shock, not to the picture of the lightning bolt.”

The questions that appeared were “How much fear did you experience when looking at this figure?” (self-reported CS fear) and “To what extent did you expect an electrical shock while looking at this figure?” (US expectancy). Participants responded through clicking one of the numbers of a 9-point Likert scale (with *1* = none at all/certainly not; *3* = very little/rather not; *5 = *uncertain; 7 = quite some/to some extent; *9* = very much/most certainly) using the computer mouse. Numbers 1, 3, 5, 7, and 9 of this scale carried a response label that was presented right above the number.

Participants indicated US valence and pain on similar 9-point Likert scales. The questions here asked “To what extent did you like the shock?” (valence); and “To what extent did you experience the shock as painful?” (pain).

#### Electrodermal responding

Specific SCRs were recorded with a Coulbourn Lablinc V, which was gated to a PC through a Scientific Solutions DMA card. Signals were digitized through customized software (Psychophysiological Recording; PSPHR). Skin conductance was recorded with standard Ag/AgCl electrodes (0.8 cm diameter) filled with KY-jelly. The electrodes were attached on the thenar and hypothenar eminences of the non-preferred hand, which was first cleaned with tap water. The signal was measured using a constant voltage (0.5 V) coupler, and digitized at 10 Hz.

The recorded data were analyzed off-line with Psychophysiological Analysis (PSPHA) [27]. For each trial, SCR (in µS) was calculated by subtracting the mean value of a baseline period (2 s before CS onset) from the highest amplitude in a 1–8 s time window after CS onset [28]. To account for individual differences in response range [29], amplitudes were range corrected using the largest measured response for that participant during the entire experiment. Finally, in order to normalize the data [30], range corrected amplitudes were square root transformed prior to further analysis.

#### Questionnaires

All participants completed the trait version of the State-Trait Anxiety Inventory (STAI-T; [31]; Dutch translation: [32]). This questionnaire assesses the disposition to feel anxious and tense through 20 items that are scored on a 4-point Likert scale.

A short self-made questionnaire that assessed clarity and face validity of the instructions and demand awareness was administered, but only to the second half of our sample. These sixteen participants were asked to indicate to what extent they thought the instructions had been clear and credible on a scale ranging from *0* (not at all) to *10* (very much so). In addition, they were asked to indicate whether there had been any particular instruction that they thought was less believable than the others. Then, they were asked to write down the putative goal of the study, to indicate whether they had considered the goal during the experiment itself and whether they thought this had affected their performance or responding in any way.

### Procedure

#### Preparation

Upon arrival, participants were asked to read and sign the informed consent form. All participants did so. Afterwards, they were asked to complete the STAI-trait version [31]. They were then taken to the experiment room and asked to wash their hands with tap water. When they were seated in front of the CRT screen on which the experiment was to be presented, the experimenter attached the electrodes that would deliver the shock stimulus. The tolerance level of this stimulus was determined individually. Next, the electrodes for the measurement of skin conductance responding were attached to the non-dominant hand. The skin conductance signal was checked in the adjacent test room by asking participants to breath in and out deeply. The experimenter ensured that this was accompanied by a clear rise and fall in the skin conductance signal. If it was not, the apparatus was checked and the electrodes were reattached before continuing. When the skin conductance signal clearly responded to deep respiration, or when the experimenter had ensured that there were no technical issues explaining a lack of response, the experiment commenced.

#### Start-up

After the preparation phase, participants were warned that they would be presented with an electrical shock. This shock was presented while participants were looking at a blank screen.

After the shock presentation, participants were informed that three fractal figures (snowflakes) would appear on screen repeatedly during 8 seconds. Then, they were shown a light grey-colored slide containing the three fractals during 8 seconds. Next, they were instructed that two of the fractals would sometimes be followed by shock, whereas the other fractal would never (in capital letters) be followed by shock. Subsequently, participants were alerted that they would see two slides on which the fractal-shock contingencies would be clearly displayed. They were asked to closely attend to the contingencies. Then, a slide containing both CS^+^ fractals and the text “+ electrical shock!” was presented during 8 seconds. This was followed by the 8 second presentation of a slide containing the CS^−^ fractal and the text “This figure will never be followed by the shock”.

#### Training phase

A training session was announced. Participants were informed that this session was meant to familiarize them with the stimuli and the procedures. Instructions indicated that the training phase was very similar to the test phase that would follow, except that some of the electrical shocks would be replaced by a picture of a lightning bolt. Participants were told that this was done to prevent them from getting too many shocks before the experiment would really take off. They were asked to keep in mind that whenever a lightning bold would be presented, this meant that in the actual test phase, a real shock would occur. Then, participants were shown a slide representing the CS^+^1 (with shock) and CS^+^2 (with lightning bolt) contingencies during 8 seconds.

The last page of instructions informed participants that they would be asked to perform fear and US (shock) expectancy ratings at regular intervals during the upcoming phase. They were told that no shocks would be administered during the ratings and asked to remind the most recent encounter with the fractals while answering the questions. The experimenter remained present in the experiment room while participants read through these instructions to ensure that the instructions were read carefully and to answer any questions that participants might have. Then, participants were presented with 27 conditioning trials (9 for each CS) interspersed with blocked ratings.

Each conditioning trial started with a 4 second presentation of a fixation cross. Then, the CS^+^1/CS^+^2/CS^−^ was presented for 8 s, followed by an inter-trial interval of 13, 15, or 17 s (see Figure 1). On reinforced trials, the US or the placeholder was presented at CS^+^ offset. The US was presented for 300 ms. The placeholder remained on screen for a duration of 500 ms. The CSs were presented in “mini-blocks” (one mini-block: CS^+^1, CS^+^2, CS^−^) so that each CS had been presented once before the next mini-block started. Trial order was randomized within mini-blocks. Blocked ratings of fear and US expectancy were presented after 9, 18 and 27 conditioning trials (3, 6 and 9 mini-blocks) respectively. As such, three sub-phases were created within the training phase, each containing 3 trials of CS^+^1, CS^+^2 and CS^−^. Three out of the nine CS^+^1 and CS^+^2 were reinforced during the training phase. The first, third, and second to last presentation of the CS^+^1 was followed by the US. For CS^+^2, the first, second, and last presentation was followed by the placeholder.

Each block of ratings contained 6 ratings (two for each CS). The order of rating trials within each rating block was fully randomized. Before the start of each rating block, it was stressed that by shock (expectancy), we referred solely to real shocks (i.e., not placeholders).

#### Test phase

Participants again received on-screen instructions. They were informed that the test phase would start, meaning that all shocks would be presented for real. Participants were instructed that the test phase would evolve similarly to the training phase in all other respects.

The course of this phase was very similar to that of the test phase, with 27 trials and 3 rating blocks in between. No shocks or placeholders were presented during this phase.

#### Post-conditioning phase

Participants completed US pain and valence ratings on screen. Then, electrodes were removed and participants were asked to fill out the self-made questionnaire using paper and pencil. Participants were fully debriefed at the end of the experiment.

### Data-analysis

To examine the effect of merely instruction-based versus instruction- plus experience-based conditioning on *self-reported CS fear*, *US expectancy* and *SCRs*, linear mixed effects (LME) model analyses were performed as implemented in the R package lme-4 (R Development Core Team, 2011). Mean SCRs were used for each sub-phase of conditioning between ratings (e.g., CS^+^1 training1: mean value for the three CS^+^1 trials in the first sub-phase of the training phase).

For each model, we defined as fixed effect variables the effect-coded factors *phase* (training1, training2, training3, test1, test2, test3) and *CS* (CS^−^, CS^+^1, CS^+^2) and their two-way interaction. The grouping variable *participant* was considered as random factor. For each model, we decided if by-participant random slopes for *phase* and *CS* were additionally needed using REML-based likelihood ratio tests [33]. For all three models, better model fits were obtained by including these by-participants random slopes for *phase* and *CS*. The reported p-values for the fixed effects are based on Type III ANOVA using a χ^2^-distribution.

Generalized Wald tests on the variance/covariance matrices were used to test our a priori hypotheses on the response patterns for CS^+^1 vs. CS^+^2 (instruction- plus experienced-based vs. merely instruction-based conditioning), and on the patterns for CS^−^ vs. CS^+^2 (no conditioning vs. merely instruction-based conditioning). We contrasted the following conditions for each of our three outcome variables: the estimated difference in mean SCR, self-reported fear and US expectancy between CS^+^1 and CS^+^2 averaged over the three training sub-phases, between CS^+^1 and CS^+^2 averaged over the three test sub-phases and between CS^+^1 and CS^+^2 for test1, test2 and test3 separately. Finally, we contrasted the CS^−^ and CS^+^2 conditions averaged over the three training sub-phases and averaged over the three test sub-phases. Alpha was set at .05 for all statistical tests.

Due to a technical error, self-reported fear and US expectancy ratings were not collected during training3 for 9 participants. However, by testing LME models, we could account for these missing values without losing substantial data. Additional analyses were carried out to examine whether the results were influenced by participants' trait anxiety or by their judgment on the clarity and credibility of the instructions.

## Results

### Questionnaires

The mean STAI trait score of the sample was 38.19 (*SD* = 9.47) and ranged between 22 and 59. Participants (*n* = 16) who completed the self-made questionnaire about instructions and demand awareness judged the instructions as clear and credible. On a scale of 0 to 10, the mean clarity score was 9.50 (*SD* = 0.82, range 7–10) and the mean credibility score was 8.50 (*SD* = 1.83). There was one participant only who judged credibility as low (score of 3). All other scores ranged between 7 and 10. Four participants indicated that there was one instruction they found less credible than the others. Two of these specified that they ‘did not believe that electrical stimuli would follow after some stimuli’ but did not specifically indicate which stimuli.

Nine of the 16 participants who answered the self-made questionnaire assumed that examining the association between expectation of electrical stimuli and (physiological) responding was the goal of the experiment. Some mentioned the term conditioning. One (other) participants mentioned the goal ‘to examine fear for stimuli that never really coincide with the electrical stimulus’. However, none of the participants described the real goal of experiment. Still, eleven participants indicated that during the experiment they had been thinking about its goal and seven of these mentioned that this (thinking about the goal) might have influenced their performance.

Initial analyses were performed to investigate the effects of trait anxiety, instruction clarity and instruction credibility. We used a median split procedure to investigate their effects. These factors were all were investigated through separate analyses. The analyses showed that neither trait anxiety nor instruction ratings interacted with other factors in any of the important analyses. One exception was a significant effect of instructions credibility at the start of the training phase. Participants with low credibility scores initially reported more fear and displayed higher US expectancy for the CS^+^2 relative to people with high credibility scores (*p*<.005). However, this effect was observed only in the first training sub-phase. No other effects of instruction credibility were observed. Trait anxiety, instruction clarity and credibility were therefore removed from further analyses.

### US ratings

The US was rated on Likert scales ranging from 1 to 9, with higher scores indicating more positive valence and higher painfulness. The US was rated as moderately negative (*M* = 3.72, *SD* = 1.84), but not as painful (*M* = 4.88, *SD* = 2.25).

### Subjective ratings

#### Self-reported fear

**Figure 2:**
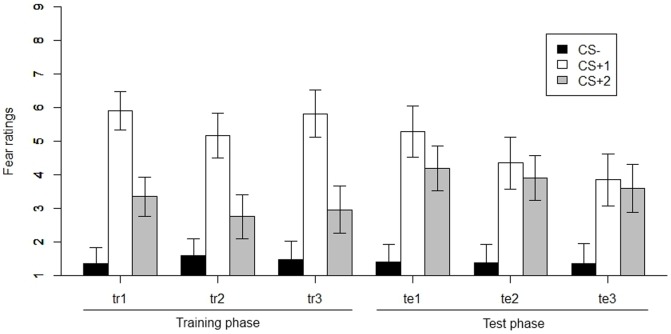
Fear ratings for CS^−^, CS^+^1 and CS^+^2 across all experimental phases (tr = training, te = test). Error bars represent the 95-percent point-wise confidence interval.

Significant main effects of both phase (χ^2^(5) = 19.50, *p*<.005) and CS (χ^2^(2) = 149.80, *p*<.001) and a significant phase x CS interaction (χ^2^(10) = 117.28, *p*<.001) were found. The estimated difference in mean fear ratings between CS^+^1 and CS^+^2 averaged over the three training sub-phases was significant (χ^2^(1) = 120.34, *p*<.001), with higher levels of reported fear for CS^+^1 than for CS^+^2 (see Figure 2). When averaging across the three test sub-phases, the estimated difference in mean fear ratings between CS^+^1 and CS^+^2 was also significant, *χ* ^2^(1) = 6.58, p = .01. During test, CS^+^1 again elicited higher self-reported fear than the CS^+^2. However, further analyses indicated that this difference was due mainly to the difference in fear ratings on test1, χ^2^(1) = 12.60, *p*<.001. That is, no significant differences in responding to CS^+^1 and CS^+^2 were observed on test2, χ^2^(1) = 2.02, *p* = .16, or test3, χ^2^(1)<1, *p* = .42 (see Figure 2). Participants reported more fear for the CS^+^2 than for the CS^−^ during the three training sub-phases (averaged), χ^2^(1) = 28.41, *p*<.001, and during the three test sub-phases (averaged), χ^2^(1) = 78.47, *p*<.001 (see Figure 2).

#### US expectancy

**Figure 3:**
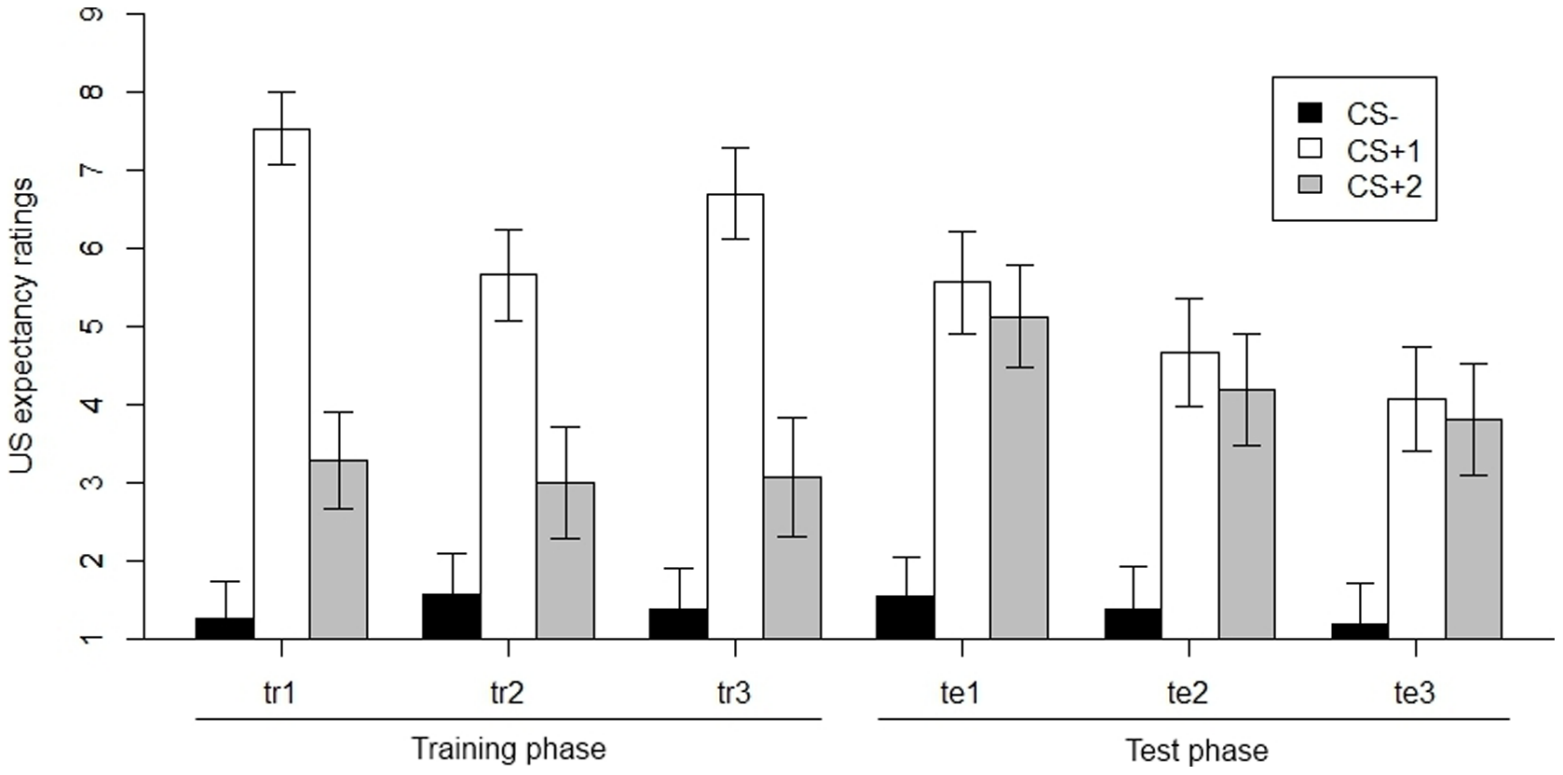
US expectancy ratings for CS^−^, CS^+^1 and CS^+^2 across all experimental phases (tr = training, te = test). Error bars represent the 95-percent point-wise confidence interval.

We observed significant main effects of phase, χ^2^(5) = 42.16, *p*<.001, and CS, χ^2^(2) = 289.87, *p*<.001, as well as a significant interaction between phase and CS, χ^2^(10) = 222.52, *p*<.001. Averaged over the three training sub-phases, CS^+^1 yielded significantly higher US expectancies than CS^+^2, χ^2^(1) = 180.71, *p*<.001 (see Figure 3). During test, in contrast, no significant difference in US expectancies for CS^+^1 and CS^+^2 was observed, χ^2^(1) = 2.30, *p* = .13. Further analyses indicated that there was no difference in mean ratings between both CS^+^s for test1, χ^2^(1) = 1.65, *p* = .20, test2, χ^2^(1) = 1.89, *p*  = .17, or test3, χ^2^(1)<1, *p* = .46. Again, the difference in responding between CS^+^2 and CS− was significant across training, χ^2^(1) = 27.19, *p*<.001, and test, χ^2^(1) = 86.03, *p*<.001, with CS^+^2 yielding higher US expectancies than CS^−^ (see Figure 3).

### SCRs

**Figure 4:**
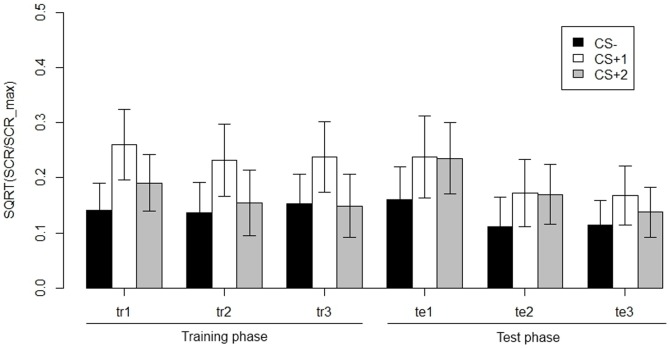
Skin conductance responses for CS−, CS^+^1 and CS^+^2 across all experimental phases (tr = training, te = test). Error bars represent the 95-percent pointwise confidence interval.

Significant main effects of both phase, χ^2^(5) = 15.82, *p* = .007, and CS, χ^2^(2) = 14.32, *p*<.001 were found. The main effect of CS confirmed the conditioning manipulation: on average, CS^+^1 elicited higher SCRs than CS^+^2 and CS−. The main effect of phase indicated that SCRs generally decreased across sub-phases (see also Figure 4). The interaction between phase and CS was not significant, χ^2^(10) = 10.70, *p* = .38. Still, when the estimated difference in mean SCR between CS*^+^*1 and CS*^+^*2 was examined separately for training (average of three training sub-phases) and test (average of three test sub-phases), we found a significant difference in SCR for CS*^+^*1 and CS*^+^*2 during training, χ^2^(1) = 13.46, *p*<.001, but not during test, χ^2^(1)<1, *p* = .57. As can be seen from Figure 4, SCRs were larger for CS*^+^*1 than for CS*^+^*2 during training, whereas the CS*^+^*s yielded similar SCRs during test. Specific contrasts for CS*^+^*1 and CS*^+^*2 for each of the test sub-phases separately revealed no differences in responding to these stimuli, all χ^2^(1)<1, all *p*'s>.34.

CS^+^2 and CS^−^ elicited similar SCRs during training (average of three training sub-phases), χ^2^(1) = 1.33, *p* = .24. During test (average of three test sub-phases), SCRs were significantly larger for CS^+^2 than for CS^−^ , χ^2^(1) = 7.78, *p*<.01 (see Figure 4).

### Comparing the effect of experience on self-reported fear, US expectancy, and SRC

To test whether self-reported fear, US expectancy, and SCR were differentially affected by experience, we computed standardized values for these three dependent variables and entered the scores obtained for the first test phase into a 3 (CS: CS−, CS^+^1, CS^+^2)×3 (type of dependent variable: self-reported fear, US expectancy, SCR) type-III ANOVA using LME models. The 3×3 ANOVA revealed significant main effects of CS (χ^2^(2) = 97.52, p<.001) and type of dependent variable (χ^2^(2) = 6.09, p<.05), as well as a significant two-way interaction between CS and type of dependent variable (χ^2^(4) = 57.69, p<.001). However, when we repeated the analysis without including the standardized SCRs, the two-way interaction between CS and type of dependent variable was not significant (χ^2^(2) = 5.32, p = .07). When we excluded the CS− data from the analysis, the two-way interaction between CS and type of dependent variable was also not significant (χ^2^(2) = 3.57, p = .17). These results indicated that interaction in the original analysis was due to the difference between the self-report measures on the one hand and SCRs on the other hand with regard to the CS− data only, indicating relatively lower values for the CS− on the self-report measures than on the SRC measure. Most importantly, the analyses did not provide any evidence for a dissociation between different dependent measures with regard to the effect of direct experience (as indexed by the difference in responding to CS+1 and CS+2). Similar analyses for comparing the effect of experience on self-reported fear, US expectancy, and SCR were performed by entering the mean scores obtained over the three test phases (instead of the scores obtained for the first test phase). Results for both analyses were similar and led to same conclusions.

## Discussion

Previous research demonstrated that mere instructions can install fear [12], [26]. Furthermore, it has been suggested that the combination of instructions and experience leads to more fear than experience alone [14]. However, the present study is the first to examine the *added* value of actual CS-US occurrences on top of instructions while controlling for CS and US presentations and the CS's predictive value. Participants were instructed that during a test phase, two stimuli (CS^+^1 and CS^+^2) would be paired with shock (US) during test. During the preceding training phase, however, only CS^+^1 was paired with the US .

The most prominent finding of our study was the fact that participants reported more fear for CS^+^1 than for CS^+^2 during the test phase. This observation provides the first demonstration of the added value of actual CS-US pairings when controlling for instructions, CS and US presentations, and the predictive value of the CS. On the other hand, no added value of experience was observed on the US expectancy and SCR measures. However, additional analyses did not reveal significant differences between the measures in the extent to which responses to CS^+^1 and CS^+^2 diverged. Although we cannot conclude that different measures are differentially sensitive to the effect of actual CS-US pairings, we can conclude that at least the fear measure is sensitive to those effects.

Before we consider the theoretical implications of the added effect of experience on self-reported fear, we need to consider the possibility that this effect was the result of demand compliance. Although self-reports are highly susceptible to demand compliance, we see at least two reasons why a demand explanation is unlikely to hold. First, it is unclear why participants would perceive a demand to report increased fear for CS^+^1 during test. Instructions clearly informed the participants that during test, the probability for the US would be exactly the same for CS^+^1 and CS^+^2. Hence, if anything, participants should perceive a demand to report equal fear for both CSs. Second, even if the participants would experience a demand to respond differently to CS^+^1 than to CS^+^2, it is unclear why they would comply to this perceived demand only when reporting their fear and not when reporting their US expectancy.

The finding that self-reported fear during test was higher for CS^+^1 than for CS^+^2 can be explained by current models of associative learning, provided that auxiliary assumptions are made. First, conditioning models of anxiety disorders can accommodate this finding but only if it is assumed that actual CS-US pairings strengthen the CS-US association that had been developed earlier on the basis of instructions [14], [16], [18]. Second, from the perspective of propositional models, enhanced self-reported fear for the CS^+^1 could indicate that direct experience is able to boost the truth value of a proposition that is already very believable based on instructions. Alternatively, it might be that the addition of any supplementary source (e.g., experience, prior knowledge, …; [23]) adds to the truth value of an existing proposition, given that this additional source provides information that confirms the nature and truth value of this proposition. In any case, our study highlights the fact that all existing models of associative learning are underspecified when it comes to dealing with the specific effects of experience and instruction. As such, our findings impose important novel constrains on any future model that attempts to deal with these issues.

Although our study provides the first evidence for an effect of CS-US pairings over and above the effect of instructions about those pairings, one might argue that the added effect of experience was actually rather limited. However, there are a number of reasons why our design might have led to an underestimation of the effect of experience. First, responses to CS^+^1 might have reached an asymptotic level during the training phase as a result of CS-US contingency instructions at the start of training. Participants rated these instructions as clear and credible. It is therefore possible that CS^+^1 responses during training reflected the effect of CS-US instructions, meaning that there was no more room for a further increase in responding based on experience. Within this line of reasoning, similar responses to CS^+^1 and CS^+^2 are to be expected during test because at that point instructions were the same for both CS^+^s. Note, however, that self-reported fear and US expectancy ratings did not approach the maximum score of 9 at any point in time. Also, the amplitude of SCRs in response to US only trials at the beginning (announced shock: *M* = .54, *SD* = .28) and at the end (unannounced shock: *M* = .80, *SD* = .31) were significantly larger than CS^+^1 SCRs (e.g., at the end of the practice phase: *M* = .24, *SD* = .20), *t*'s>4.80, *p*'s<.001, which suggest that also on this measure there was room for further increase in responding.

Second, responding to CS^+^2 might have reflected not only instructions but also experience. More specifically, the pairing of the CS^+^2 with a placeholder might have installed some level of fear conditioning in response to this stimulus, which is confirmed by elevated self-reported fear and US expectancy ratings for this stimulus relative to the CS^−^ during training. On a related note, the results might at least partially depend on the occurrence of phenomena such as US rehearsal [34] and/or sensory preconditioning with US inflation [35]. Previous research has shown that mental rehearsal of the US can maintain conditioned responding to the CS [34]. In case of the present study, the placeholder might have served as a type of US rehearsal, which might have exacerbated responding to the CS^+^2 during test, when it was instructed that the US would now be presented for real. Alternatively, a combination of sensory preconditioning and US inflation might have augmented CS^+^2 responding during test. As shown by White and Davey [35], the pairing between a CS and an innocuous US (in this case the placeholder) can lead to strong conditioned responding after the aversiveness of the US has been inflated. In the current study, US inflation might have occurred repeatedly through the shock US presentations after CS^+^1 during training and through the instructions that announced that all USs would be presented for real at the start of the test phase. We believe that the possible role of US rehearsal or sensory preconditioning with US inflation does not invalidate the claim that fear conditioning can result from instructions in the absence of actual CS-US pairings. Instead, these phenomena might explain *how* instructions can have this effect. Hence, it would be a fruitful pathway for future research to look further into the influence of these phenomena on instructed conditioning. Most importantly for the present purposes, however, it is possible that the effects of experience will be bigger in future studies that do not employ a placeholder.

There is also a third reason why the effect of experience might have been underestimated. The presentation of written instructions on a computer screen in the context of an experiment probably conveys high credibility and gives little reason to doubt the validity of the instructions. We did indeed observe that participants were confident that the instructions were correct. Assuming that the effect of instructions depends on the credibility of those instructions, it is thus likely that we maximized the effect of the instructions, therefore leaving less room for an additional effect of experience.

Finally, the null effects of experience that were observed on the US expectancy and SRC measures could have been due to a lack of statistical power. Post-hoc power analyses showed that our tests had sufficient power to detect medium sized effects (Cohen's d of .50) but not small effects (Cohen's d of .20).

Whereas these four arguments call for caution when interpreting the similarities between the effects of experience and instructions, they add weight to the observation that experience did have an impact on self-reported fear. Because we observed this effect despite a variety of factors that could have counteracted the effect of experience, we can be quite confident in our conclusion that experience does contribute to fear conditioning over and above the impact of instructions.

Apart from revealing differences and similarities between the effects of experiences and mere instruction, our study also confirmed that a CS does not have to be paired with the actual US in order to observe fear conditioning. This observation confirms previous findings and has important clinical implications (see Field, 2006 [22], for a discussion on conditioning in the absence of CS and US). With regard to prevention, this signals the need to inform parents and teachers on this pathway to fear. It might be that people intuitively underestimate the impact of threatening verbal statements or warnings towards children relative to the actual experience of a threatening event. On the other hand, our study shows that the experience of CS-US pairings can have effects over and above the effect of instructions.

One unique aspect of the current study in comparison to previous studies on fear conditioning via instructions is that we also gave information about when instructed CS-US relations would actually occur. More specifically, participants were told that CS^+^2 would be followed by the actual US only during the test phase. Interestingly, the relative difference in self-reported fear for CS^+^2 compared to CS− was larger at the start of the test phase than at the end of the training phase. In other words, the effect of instructions about CS-US parings became stronger at the time that the pairings were said to occur. This implies that the effect of CS-US instructions is not merely due to pairing symbolic representations of the CS and the US during the instructions (i.e., the mere co-occurrence of words referring to the CS and US in verbal sentences; see [22]). The effect also seems to depend on the actual meaning of the instructions, that is the fact information conveyed in the instruction about when the pairings will actually occur. Note, however, that during training, we also observed conditioned responding to CS^+^2 despite the fact that the instructions clearly specified that CS^+^2 could not be followed by the US during that phase. It is not clear whether this effect is due to the mere pairing of CS^+^2 with a symbolic representation of the US (either the word “shock” in the instructions or the placeholder) or to the fact that participants somewhat distrusted the validity of instruction that CS^+^2 would not be followed by the US during the training phase.

Finally, we also want to point to a limitation of our study. Because we used a within subjects design, all participants experienced the US. We therefore do not know whether our results generalize to situations in which the US is never presented. For instance, it is possible that threat information or warnings in real-life situations lead to fear responses only when the US information refers to a situation that has actually been experienced, or, as suggested by Davey [18], when the threat information US is revalued at some point (US inflation). It would, therefore, be interesting for future research to replicate the present study with a between-subjects design, in which the instructed group is never actually presented with the relevant US.

In sum, the present study illustrates that, at least with regard to self-reported fear, actual CS-US pairings add to the effect of clear instructions on the CS-US contingencies. Our study thus opens up a new line of research about when and how experience has effects over and above the effects of instructions. A fruitful option would be to add dependent variables that could shed some light on the conditions under which actual CS-US pairings add to the effect of instructions.

## References

[1] Rachman S (1977) Conditioning Theory of Fear-Acquisition - Critical-Examination. Behaviour Research and Therapy 15: 375–387. 612338 10.1016/0005-7967(77)90041-9

[2] Muris P, Merckelbach H, Collaris R (1997) Common childhood fears and their origins. Behaviour Research and Therapy 35: 929–937. 9401133 10.1016/s0005-7967(97)00050-8

[3] Muris P, du Plessis M, Loxton H (2008) Origins of common fears in South African children. Journal of Anxiety Disorders 22: 1510–1515. 18417318 10.1016/j.janxdis.2008.03.004

[4] Ollendick TH, King NJ (1991) Origins of Childhood Fears - an Evaluation of Rachman Theory of Fear Acquisition. Behaviour Research and Therapy 29: 117–123. 2021373 10.1016/0005-7967(91)90039-6

[5] King NJ, Eleonora G, Ollendick T (1998) Etiology of childhood phobias: current status of Rachman's three pathways theory. Behaviour Research and Therapy 36: 297–309. 9642849 10.1016/s0005-7967(98)00015-1

[6] Askew C, Field AP (2008) The vicarious learning pathway to fear 40 years on. Clinical Psychology Review 28: 1249–1265. 18614263 10.1016/j.cpr.2008.05.003

[7] Muris P, Field AP (2010) The Role of Verbal Threat Information in the Development of Childhood Fear. “Beware the Jabberwock!”. Clinical Child and Family Psychology Review 13: 129–150. 20198423 10.1007/s10567-010-0064-1 PMC2882043

[8] Field AP, Argyris NG, Knowles KA (2001) Who's afraid of the big bad wolf: a prospective paradigm to test Rachman's indirect pathways in children. Behaviour Research and Therapy 39: 1259–1276. 11686263 10.1016/s0005-7967(00)00080-2

[9] Field AP, Lawson J (2003) Fear information and the development of fears during childhood: effects on implicit fear responses and behavioural avoidance. Behaviour Research and Therapy 41: 1277–1293. 14527528 10.1016/s0005-7967(03)00034-2

[10] Lawson J, Banerjee R, Field AP (2007) The effects of verbal information on children's fear beliefs about social situations. Behaviour Research and Therapy 45: 21–37. 16529711 10.1016/j.brat.2006.01.007

[11] Field AP, Schorah H (2007) The verbal information pathway to fear and heart rate changes in children. Journal of Child Psychology and Psychiatry 48: 1088–1093. 17995484 10.1111/j.1469-7610.2007.01772.x

[12] Olsson A, Phelps EA (2004) Learned fear of “unseen” faces after Pavlovian, observational, and instructed fear. Psychological Science 15: 822–828. 15563327 10.1111/j.0956-7976.2004.00762.x

[13] Cook SW, Harris RE (1937) The verbal conditioning of the galvanic skin reflex. Journal of Experimental Psychology 21: 202–210.

[14] Field AP, Storksen-Coulson H (2007) The interaction of pathways to fear in childhood anxiety: A preliminary study. Behaviour Research and Therapy 45: 3051–3059. 17935694 10.1016/j.brat.2007.09.001

[15] Ugland CC, Dyson BJ, Field AP (2013) An ERP study of the interaction between verbal information and conditioning pathways to fear. Biol Psychol 92: 69–81. 22366224 10.1016/j.biopsycho.2012.02.003

[16] Davey GCL (1997) A conditioning model of phobias. In: Davey GCL, editor. Phobias: A handbook of theory, research and treatment. Chichester: Wiley. pp. 301–322.

[17] Mineka S, Zinbarg R (2006) A contemporary learning theory perspective on the etiology of anxiety disorders - It's not what you thought it was. American Psychologist 61: 10–26. 16435973 10.1037/0003-066X.61.1.10

[18] Davey GCL (1992) Classical-Conditioning and the Acquisition of Human Fears and Phobias - a Review and Synthesis of the Literature. Advances in Behaviour Research and Therapy 14: 29–66.

[19] Dickinson A, Burke J (1996) Within-compound associations mediate the retrospective revaluation of causality judgements. Quarterly Journal of Experimental Psychology Section B-Comparative and Physiological Psychology 49: 60–80. 10.1080/713932614 8901386

[20] Rescorla RA, Wagner AR (1972) A theory of Pavlovian conditioning: Variations in the effectiveness of reinforcement and non-reinforcement. In: Black AH, Prokasy WF, editors. Classical conditioning: Current theory and research. 1 ed. New York: Appleton-Century-Crofts. pp. 64–99.

[21] Wagner AR (1981) SOP: A model of automatic memory processing in animal behavior. In: Spear NE, Miller RR, editors. Information processing in animals: Memory mechanisms. Hillsdale, NJ: Erlbaum. pp. 5–47.

[22] Field AP (2006) Is conditioning a useful framework for understanding the development and treatment of phobias? Clinical Psychology Review 26: 857–875. 16472895 10.1016/j.cpr.2005.05.010

[23] De Houwer J (2009) The propositional approach to associative learning as an alternative for association formation models. Learning & Behavior 37: 1–20. 19122048 10.3758/LB.37.1.1

[24] Mitchell CJ, De Houwer J, Lovibond PF (2009) The propositional nature of human associative learning. Behavioral and Brain Sciences 32: 183–+. 19386174 10.1017/S0140525X09000855

[25] Miller RR, Escobar M (2001) Contrasting acquisition-focused and performance-focused models of acquired behavior. Current Directions in Psychological Science 10: 141–145.

[26] Field AP, Lawson J (2008) The verbal information pathway to fear and subsequent causal learning in children. Cognition & Emotion 22: 459–479.

[27] De Clercq A, Verschuere B, De Vlieger P, Crombez G (2006) Psychophysiological Analysis (PSPHA): A modular script-based program for analyzing psychophysiological data. Behavior Research Methods 38: 504–510. 17186761 10.3758/bf03192805

[28] Pineles SL, Orr MR, Orr SP (2009) An alternative scoring method for skin conductance responding in a differential fear conditioning paradigm with a long-duration conditioned stimulus. Psychophysiology 46: 984–995. 19558401 10.1111/j.1469-8986.2009.00852.x PMC2868319

[29] Lykken DT, Venables PH (1971) Direct Measurement of Skin Conductance - Proposal for Standardization. Psychophysiology 8: 656–&. 5116830 10.1111/j.1469-8986.1971.tb00501.x

[30] Dawson ME, Schell AM, Fillion DL (2007) The electrodermal system. In: Cacioppo JT, Tassinary LG, Bernston GG, editors. Handbook of Psychophysiology. third ed. Cambridge: Cambridge University Press. pp. 159–181.

[31] Spielberger CD, Goruch RL, Lushene R, Vagg PR, Jacobs GA (1983) Manual for the State-Trait Anxiety Inventory. Palo Alto, CA: Consulting Psychologists Press.

[32] van der Ploeg HM, Defares PB, Spielberger CD (2000) Handleiding bij de Zelfbeoordelings Vragenlijst. Een Nederlandstalige bewerking van de Spielberger State-Trait Anxiety Inventory [Manual for the State-Trait Anxiety Inventory. A Dutch translation]. Lisse, The Netherlands.

[33] West BT, Welch KB, Galecki AT (2007) Linear mixed models: A practical guide using statistical software. London: Chapman & Hall/CRC.

[34] Jones T, Davey GCL (1990) The Effects of Cued Ucs Rehearsal on the Retention of Differential Fear Conditioning - an Experimental Analog of the Worry Process. Behaviour Research and Therapy 28: 159–164. 2327933 10.1016/0005-7967(90)90028-h

[35] White K, Davey GCL (1989) Sensory Preconditioning and Ucs Inflation in Human Fear Conditioning. Behaviour Research and Therapy 27: 161–166. 2930441 10.1016/0005-7967(89)90074-0

